# Health Tract, No. 192

**Published:** 1865

**Authors:** 


HEALTH TRACT, No. 192.

---

### MIND AND BODY.

THE influence which the mind has in causing, aggravating, and protracting disease, is too constantly lost sight of, by all classes of physicians. Every body recommends exercise as a means of preserving and regaining health. But to ride a certain length of time, or to walk a specified distance "for the health," merely for the sake of the health, is almost useless, and is a penance; but if there is the accompaniment of an agreeable associate or an exhilarating motive, one which lifts up the mind and absorbs it for the time being, so as to make it wholly forgetful of the bodily condition, as the radical object of the exercise, this is health giving; its effects are always magical, on mind and body and blood.

Dwelling on trouble; remorse for lost opportunities; the hugging of sharp-pointed memories; moping over the slights of friends; feeding on exaggerations of the hardness of our lot, and grieving vainly for unrequited love, all these are known the world over, as being capable of bringing on slow and painful and fatal diseases. But it is not so well understood that great mental emotion sometimes causes maladies which prove fatal in a few days; such maladies as are induced by great physical exposures. It was recently announced that a distinguished French advocate was so excited and exhausted by one of his professional efforts, as to superinduce an attack of pneumonia, (lung fever or inflammation of the lungs,) of which he died in a few days. Three young ladies were riding in a carriage in St. Louis; the horses ran away; two of the riders escaped from the vehicle, while the third sat still, as composedly as if nothing unusual had taken place; all were astonished at her "presence of mind." After she reached her home, she informed her friends that she remained still because the shock, the feeling of horror was such, that she was per force, as immovable as marble; the reäction was such as to cause an inflammation of the bowels, which nothing could remove, and of which she died in a few days. These facts, with thousands of others like them, prove beyond all cavil, that the mind may be a cause of disease; and the inference is clear, that the states of the mind should be watched. We should guard against cherishing depressing feelings; and with as much care, should habituate ourselves to self-control; to the habit of looking at every thing of a stirring or harrowing character with a calm courage; we should strive at all times for that valuable characteristic, "presence of mind," under all circumstances; for we are every day in great need of it; it is in many cases, a literal "life-preserver."

